# Exposure to maternal obesogenic diet worsens some but not all pre-cancer phenotypes in a murine genetic model of prostate cancer

**DOI:** 10.1371/journal.pone.0175764

**Published:** 2017-05-10

**Authors:** Theresa Okeyo-Owuor, Emily Benesh, Scott Bibbey, Michaela Reid, Jacques Halabi, Siobhan Sutcliffe, Kelle Moley

**Affiliations:** 1 Department of Obstetrics and Gynecology, Washington University School of Medicine, Saint Louis, Missouri, United States of America; 2 Department of Pathology and Immunology, Washington University School of Medicine, Saint Louis, Missouri, United States of America; 3 Department of Surgery, Washington University School of Medicine, Saint Louis, Missouri, United States of America; National Cancer Institute, UNITED STATES

## Abstract

Prostate cancer research has been predominantly focused on adult exposures and risk factors. However, because the prostate develops during gestation and early life, exposure to external factors, such as obesity, during development could affect the prostate cancer progression in adults. Our previous work demonstrated that exposure to a high fat/high sugar (HF/HS) diet during gestation and until weaning stimulated prostate hyperplasia and altered the Pten/Akt pathway in adult mice fed a normal diet after weaning. Here, we asked whether maternal exposure to HF/HS would worsen prostate phenotypes in mice lacking Pten, a widely accepted driver of prostate cancer. We found that, at six weeks of age, both Chow (control)—and HF/HS-exposed Pten knockout mice showed evidence of murine PIN that included ducts with central comedo necrosis but that the HF/HS exposure did not influence murine PIN progression. The Pten knockout mice exposed to HF/HS *in utero* had significantly more mitotic cells than Pten knockouts exposed to Chow diet. In the Pten null background, the maternal HF/HS diet enhanced proliferation but did not have an additive effect on Akt activation. We observed neuroendocrine differentiation in Pten knockout mice, a phenotype that had not been previously described in this model.

## Introduction

Prostate cancer is the most commonly diagnosed male cancer in the western world. It is the second leading cause of death in men in the United States (behind lung cancer) with 180,890 new cases and 21,120 deaths projected for 2016[1]. Few modifiable risk factors exist for this disease but may include obesity, particularly for advanced disease[2] [3]. Because the human prostate develops *in utero* and during early life, it was recently proposed that adverse exposure to carcinogens during those time periods could influence adult prostate health[4]. Another *in utero* exposure that could influence prostate cancer risk is maternal obesity, which affects 36% of women in the United States in 2016[5, 6]. In support of this idea, higher gestational levels of steroid hormones and growth factors, such as insulin-like growth factor 1, have been hypothesized to increase the number of prostate stem cells, which are at risk for later transformation[7]. Gestational exposures, such as estrogens and androgens, may also alter mutation rates[7, 8], directly damage fetal DNA[9], and permanently alter hormonal signaling pathways, thereby affecting prostate development, responsiveness to hormones, and cancer risk[4]. Precedent for this idea also comes from other hormone-dependent neoplasms such as breast cancer.[10] For example, maternal obesity influences mammary tumor development in rodent models, and maternal obesity in strongly correlated with breast cancer incidence in rodent offspring[11–13].

In previous work, we demonstrated that C57Bl/6J female mice exposed to a high-fat, high-sucrose (HF/HS) diet from one month before conception until weaning delivered male offspring that, as adults, had higher levels of prostate hyperplasia and nuclear atypia than offspring of control Chow-exposed mice[14]. The prostate hyperplasia was most pronounced in the oldest exposed male offspring at 63 weeks and was associated with hyperactivation of the Akt pathway through deactivation of Pten. Our previous data thus suggested that maternal obesity is a novel risk factor for initiation of prostate cancer.

Here, we investigated whether exposure to maternal obesogenic diet would act as a "second hit" to worsen the effects of genetic lesions that are important for prostate cancer development. We used a well-established Pten homozygous knockout mouse model, in which the Probasin (PB) promoter drives expression of Cre recombinase in the prostate [15, 16]. The Probasin Cre transgene is expressed postnatally in the prostate epithelium and therefore the Pten gene is deleted in these mice after birth[16]. Pten knockout male offspring exposed to Chow or HF/HS diet *in utero* and until weaning exhibited mPIN at six weeks of age. Prostates of Pten knockout mice exposed to HF/HS *in utero* had enhanced proliferation. Finally, many of the Pten knockout mice exhibited neuroendocrine differentiation (NED), a hallmark of prostate cancer progression that correlates with tumor grade and poor prognosis in humans[17].

## Materials and methods

### Animal husbandry

All animal procedures were performed in accordance with an animal protocol approved by the Animal Studies Committee at Washington University School of Medicine. 129S4-Pten^tm1Hwu^ (referred to as Pten^loxp/loxp^) female mice were obtained from Jackson Laboratories. Pten^loxp/loxp^;PB-Cre4^+^ male mice (in which the Cre recombinase is under the control of an enhanced prostate-specific Probasin promoter) were a generous gift of Dr. Helen Piwnica-Worms. Four-week-old Pten^loxp/loxp^ female mice were fed either control Chow diet (PicoLab Rodent Diet 20; 13.2% fat, 62% carbohydrates [3.2% sucrose]) or standard high-fat/high-sucrose (HF/HS) diet (TestDiet, 58R3; 59.4% fat, 26% carbohydrates [17% sucrose])[18] for a minimum of five weeks, then mated to Pten^loxp/loxp^;PB-Cre4^+^ males (previously exposed to control Chow diet). The dams were fed their respective diets throughout gestation and weaning. The male pups from both Chow and HF/HS-fed dams were fed control Chow after weaning until sacrifice at six weeks of age. Genotypying of tail DNA was carried out by using Klentaq polymerase mix (DNA Polymerase Technology). The PCR reaction was as follows: 93°C for 1 minute, then 30 cycles of 93°C for 20 seconds and 68°C for 3 minutes, then finished at 10°C. Primers used were: Pten: 5’TAA GGA AGA GGG TGG GGA TAC CAG GGA T3’ and 5’GGG CAC TTT CAC TGC TAC CCT GAG CTT T3’, Cre: 5’GCA TTA CCG GTC GAT GCA ACG AGT GAT GAG3’ and 5’GAG TGA ACG AAC CTG GTC GAA ATC AGT GCG3’. At sacrifice, urogenital sinus regions were removed en bloc to cold PBS (pH 7.4), and dorsolateral prostates (DLPs) were disassociated and processed as previously described[19, 20].

### Metabolic analysis

At nine weeks of age, dams were weighed, and body composition was analyzed by quantitative magnetic resonance imaging (EchoMRI- 900). For metabolic assessments, mice were fasted for six hours and injected intraperitoneally with 10% glucose at a dose of 1 mg/g of body weight. A glucometer (Contour TS; Bayer) was used to measure glucose from whole blood at 0, 15, 30, 60, 90 and 120-minute intervals. Fasting serum insulin was measured with the rat/mouse insulin enzyme-linked immunosorbent assay kit (EMD Millipore) as per manufacturer’s instructions. Insulin resistance was calculated by using the Homeostatic Model Assessment test (HOMA-IR) as previously described[21].

### Hematoxylin and Eosin (H & E) staining

Prostate sections were fixed overnight in 4% paraformaldehyde and processed for histological analyses as previously described[14]. The DLP sections were stained with Hematoxylin and Eosin and analyzed by a resident expert pathologist blinded to the feeding regimen and genotype. The ducts with expansile central necrosis were quantified as a percentage of total number of ducts. The number of cells undergoing mitosis (as denoted by mitotic figures visible with a light microscope at 20X magnification) were counted per 10 high-powered fields.

### Immunohistochemistry (IHC) and immunofluorescence (IF)

Slides were processed as previously described[14, 22]. For IHC, sections were incubated with antibody specific to Synaptophysin (Sigma, 1:250) overnight at 4°C, then in goat HRP-conjugated secondary antibody (Santa Cruz Biotechnology, 1:1000). Slides were developed for 5 minutes with a Vector DAB Kit (Vector Laboratories) per manufacturer's protocol, counterstained in CAT Hematoxylin, and imaged on a Nikon Eclipse E800 upright microscope with an Olympus DP71 camera and manufacturer's software. For IF, primary antibodies against the following proteins were used: phospho-histone H3 (pHH3) (Abcam, 1:750), Ki67 (Abcam, 1:250), phospho-Akt (Ser-473), Akt and Pten (1:100, Cell Signaling) followed by Alexa Fluor 488/546/647-conjugated secondary antibodies raised in goat (Molecular Probes,1:1000). Sections were counterstained with DAPI. Imaging was performed by confocal microscopy (Leica TCS SPE/DMI4000B; Leica Application Suite software). The numbers of pHH3- and Ki67-positive cells were counted and normalized to the total number of nuclei per image.

### Statistical analyses

Data are expressed as mean ± SEM for normally distributed values and as median (range) for non-normally distributed values. Metabolic data from dams were calculated using Student’s t-test of means (n = 3–6 females). The male offspring data are from the following groups: 10 WT—HF/HS (5 litters with at 1–4 mice per litter), 9 Pten KO—HF/HS (4 litters with 1–3 mice per litter), 15 WT—CHOW (6 litters with at least 1–4 mice per litter), and 14 Pten KO—CHOW (5 litters with at least 1–7 mice per litter). All offspring data was calculated as follows: medians (or non-normal distributions) were compared by Wilcoxon rank sum test, proportions were compared by Fisher’s exact test, and means were compared using 2-way analysis of variance (ANOVA) followed by Tukey’s multiple comparison’s test. GraphPad PRISM (Version 6.0, La Jolla) and SAS (Version 9.4, Cary, NC) were used for statistical analyses. For all tests, *P* < 0.05 was considered statistically significant.

## Results

### Maternal high-fat diet does not accelerate the progression of murine prostatic interepithelial neoplasia in male offspring

**Fig 1:**
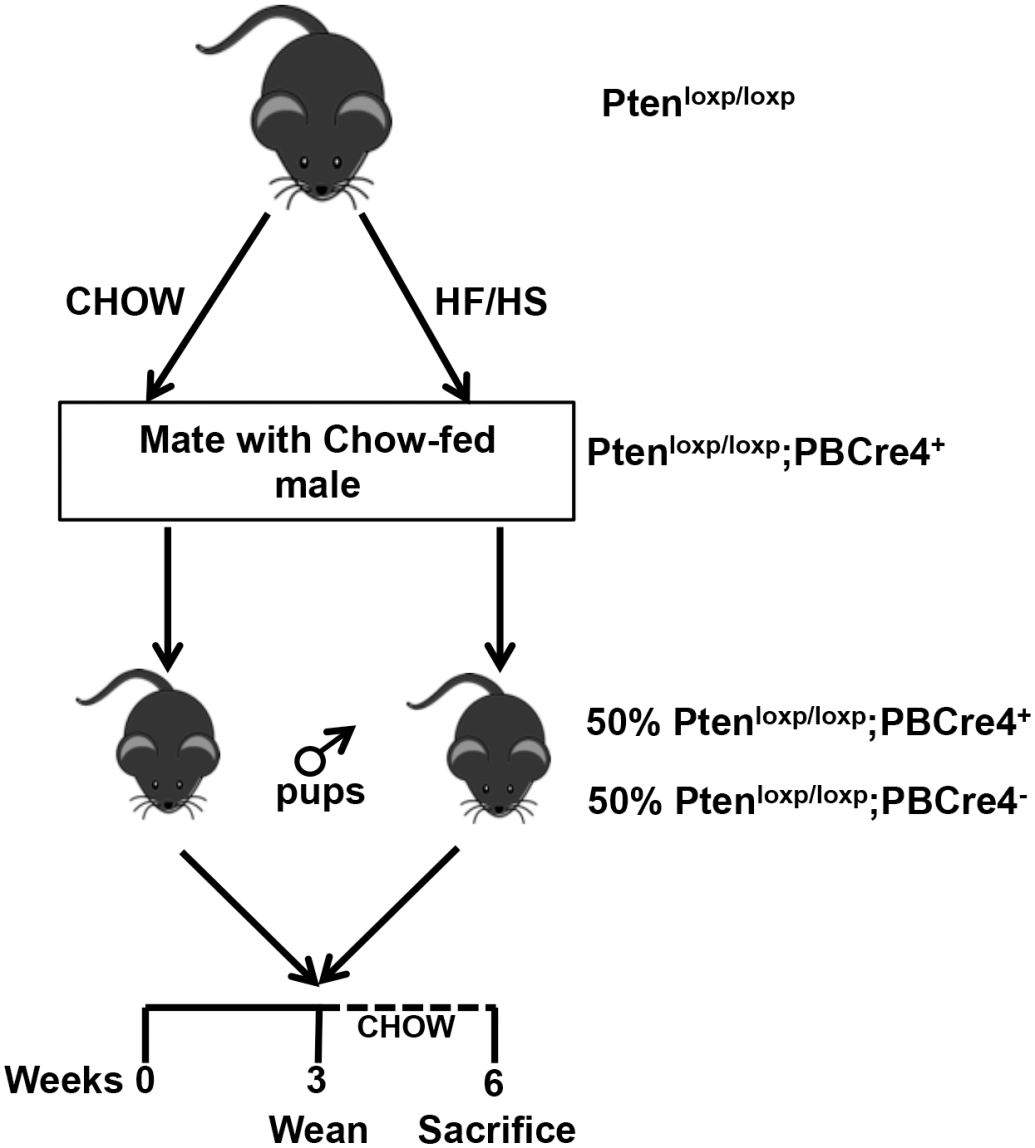
Experimental set-up. Four-week-old Pten^loxp/loxp^ female mice were fed either a Chow or HF/HS diet for four weeks and then mated to Pten^loxp/loxp^;PBCre4^+^ males. Male offspring were fed a Chow diet after weaning (three weeks) and until sacrifice at six weeks of age.

**Fig 2:**
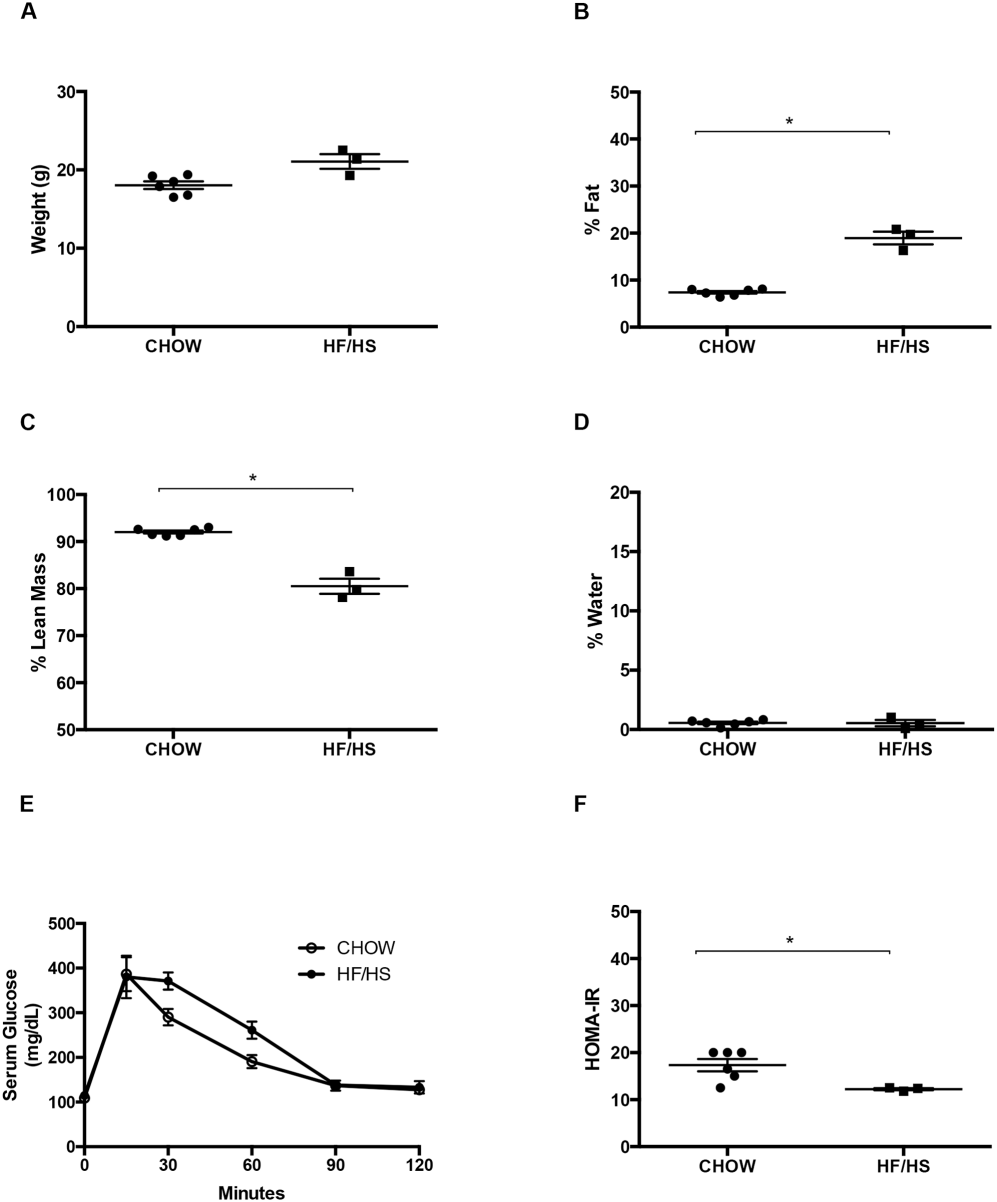
Metabolic consequences of HF/HS diet in nine-week-old Pten^loxp/loxp^ dams. Dot plots show quantitation of body weight (A), percent fat mass (B), percent lean mass (C), percent water (D), glucose tolerance (E), and insulin sensitivity (F). n > 3 mice per group, *P<0.05 as calculated by student’s t-test.

To determine the impact of maternal diet and genetic alterations on prostate cancer outcomes, we fed a Chow control or HF/HS diet to four-week-old Pten^loxp/loxp^ female mice for a minimum of five weeks. We measured the metabolic features of these F0 mice at nine weeks of age (discussed below), then mated them to Pten^loxp/loxp^;PB-Cre4^+^ male mice (Fig 1). The resulting F1 pups were either Pten^loxp/loxp^;PB-Cre4^+^ (Pten knockouts) or Pten^loxp/loxp^;PB-Cre4^-^ (wildtype [WT]) littermate controls. Their respective mothers raised all pups until weaning (at three weeks of age), at which point the pups were fed a Chow diet until sacrifice at six weeks of age (Fig 1). Metabolic analysis of the Pten^loxp/loxp^ F0 dams showed that mothers fed the HF/HS diet had a slightly but not significantly higher body weight than those fed a Chow diet (Fig 2A). The HF/HS-exposed dams had significantly higher percent fat and lower percent lean mass than Chow-exposed dams (Fig 2B and 2C). Percent water did not differ between the groups. HF/HS-exposed mice had normal glucose tolerance but were less insulin sensitive than their Chow-fed counterparts (Fig 2D–2F) (See Tables A and B in S1 File).

**Fig 3:**
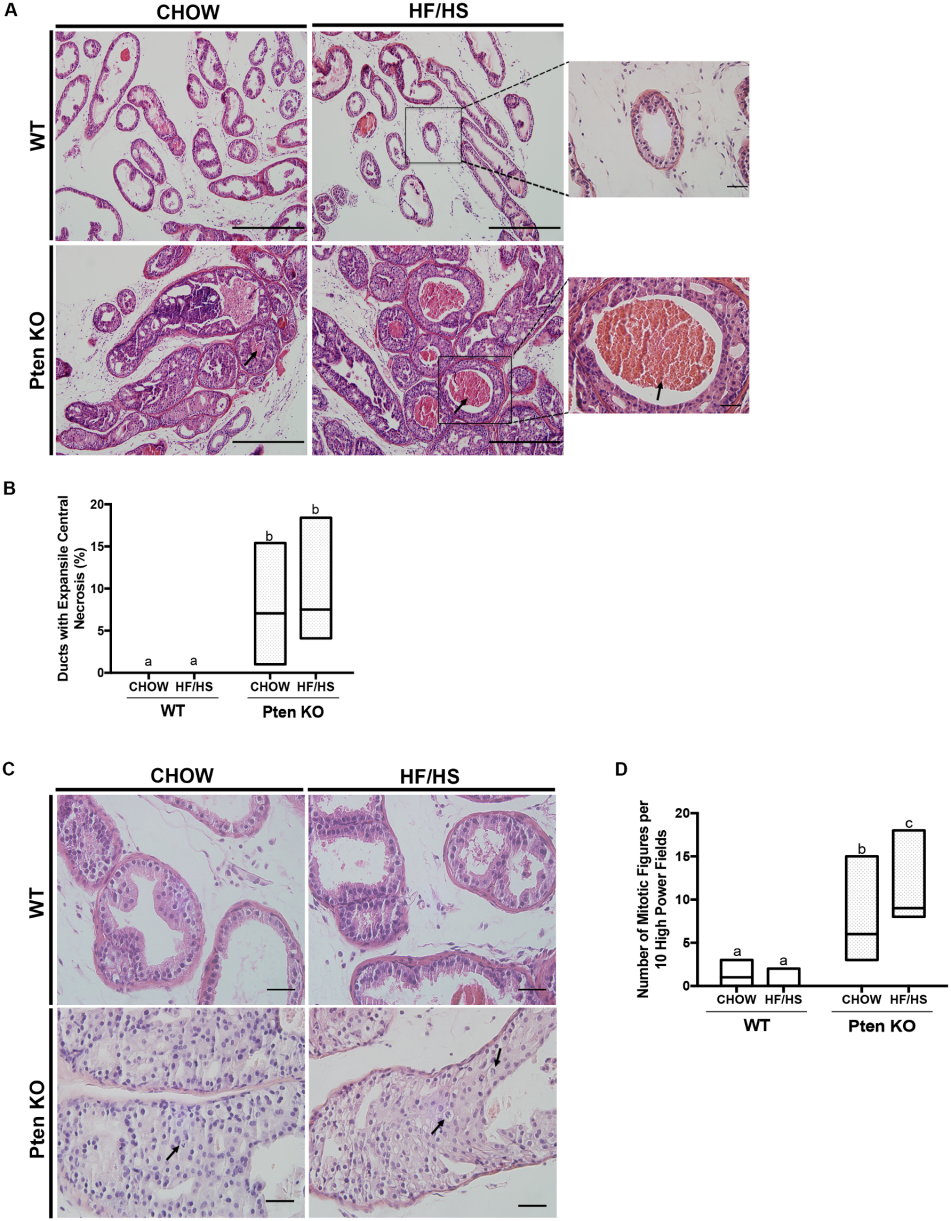
HF/HS diet exposure increases mitotic figures in prostates of Pten knockout males. (A) Representative H&E staining of dorsolateral prostates (DLPs) from six-week-old WT and Pten knockout offspring from to Chow or HF/HS-fed dams. Black arrows indicate expansile central comedo necrosis. Scale bars = 500 μm in main panel and 100 μm in inset. (B) Quantification of ducts with expansile central necrosis (% of total ducts per prostate section). (C) Representative H&E staining of DLP sections. Arrows indicate mitotic figures. Scale bars = 100 μm. (D) Numbers of prostate cells undergoing mitosis per 10 high-powered fields in Pten knockout mice exposed to Chow or HF/HS. Same letters mean there’s no significant difference between conditions, different letters represent statistically significant differences. N >10 mice; *P*<0.05 as calculated by Wilcoxon rank sum test, plotted using median and range.

Male mice of this Pten knockout genotype have been shown to develop mPIN at six weeks that progresses to invasive adenocarcinoma by 9 weeks of age[15]. In our studies, both the Chow- and HF/HS-exposed Pten knockout mice exhibited mPIN at six weeks. Specifically, prostatic ducts in the Pten knockout mice showed proliferations of large atypical epithelial cells with stratification and variable cribriform architecture (Fig 3A, lower panels), which was not observed in WT littermate controls (Fig 3A, upper panels). We also observed central comedo necrosis a feature, which has been described in intraductal carcinoma of the prostate (IDC-P) **(**Fig 3A, lower panel, black arrows; Table 1). IDC-P is an entity associated with invasive carcinoma and aggressive disease in humans[23]. When we quantified ducts with expansile central comedo necrosis by counting only those lumens with necrotic or dead cells that were noticeably expanding the duct, we found no significant difference between the Pten knockout mice born to HF/HS- or Chow-fed dams (Fig 3B, Table 1). This data suggest that exposure to maternal HF/HS diet does not have an additive effect on Pten loss in the progression of mPIN to a more advanced disease state (See S2 and S3 Files).

**Table 1:** Summary of prostate cancer phenotypes in Pten knockout mice and WT littermate controls.

Histology	Pten^loxp/loxp^;PB-Cre4^-^ (WT)		Pten^loxp/loxp^;PB-Cre4^+^ (Pten KO)	
	CHOW (n = 10)	HF/HS (n = 12)	CHOW (n = 13)	HF/HS (n = 11)
Mice with mPIN (%)	0	0	92.3*	100^$^
Mice with central comedo necrosis (%)	0	0	92.3*	100^$^

*WT versus Pten KO mice exposed to Chow; p<0.01

^$^WT versus Pten KO mice exposed to HF/HS; p<0.01

Calculated using Fisher’s exact test

### Maternal high-fat diet increases proliferation in both WT and Pten knockout male offspring

**Fig 4:**
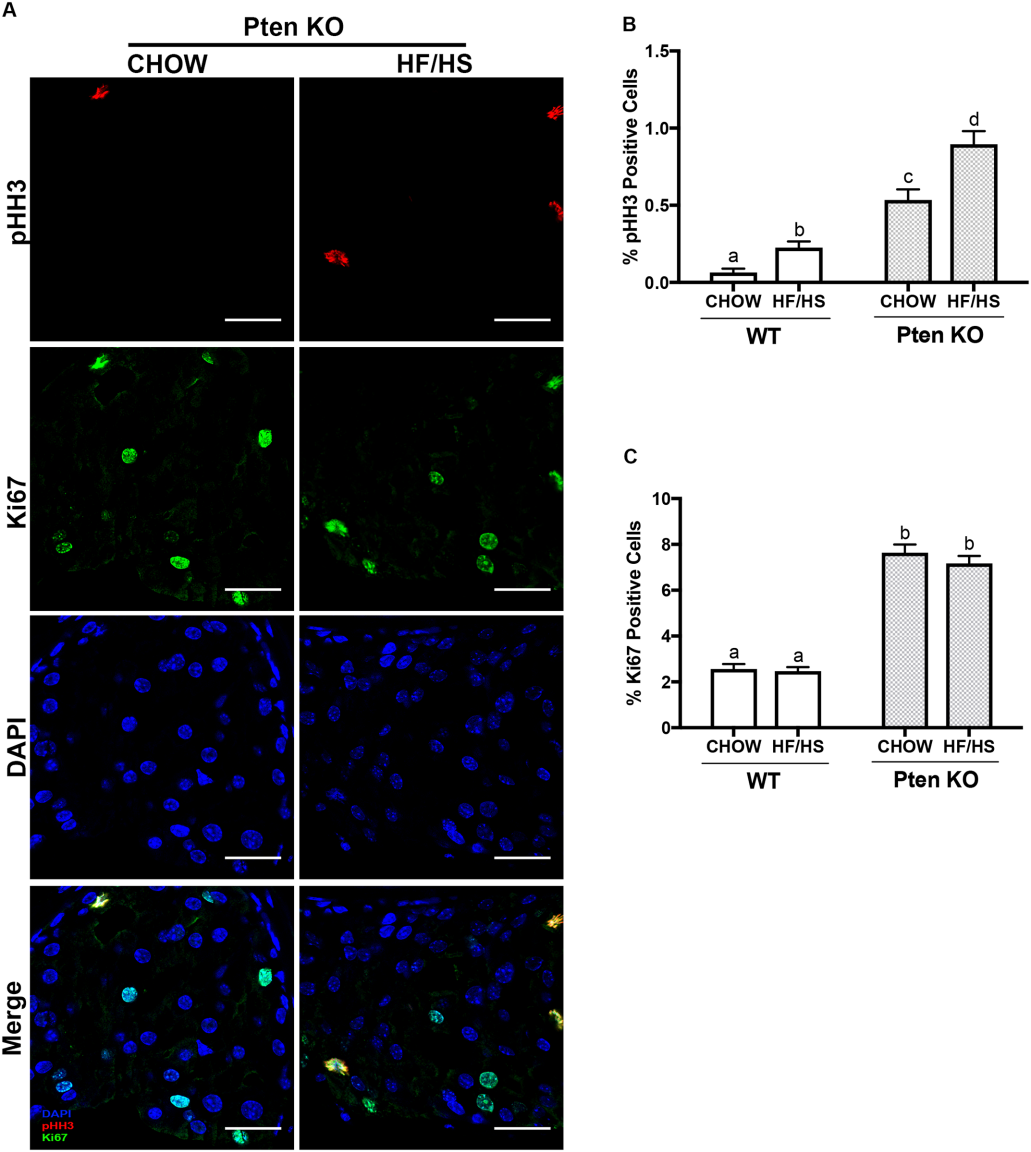
HF/HS diet exposure stimulates increased proliferation in male offspring. (A) Representative pHH3 and Ki67 staining of prostates from Pten knockout mice exposed to maternal Chow or HF/HS diet Red—pHH3, Green—Ki67, Blue—DAPI (nuclei). Scale bars = 25 μm. (B and C) Quantification of pHH3- (B) and Ki67- (C) positive cells normalized to total number of cells per image. Same letters mean there’s no significant difference between conditions, different letters represent statistically significant differences. n = 10 images per mouse from n = 5 mice per experimental group; *P*<0.05 as calculated by 2-way analysis of variance followed by Tukey’s multiple comparison’s test.

In our histological analyses, we noticed that the Pten knockout offspring of HF/HS-fed dams had more prostatic cells undergoing mitosis than did Pten knockout offspring of Chow-fed dams (Fig 3C, black arrows**)** (Chow 6[3–15] vs. HF/HS 9[8–18], median [range], p<0.01 (Fig 3D)). Pten WT results are summarized in Fig 3D. To confirm this finding, we assessed the expression of phospho-histone H3 (pHH3) and Ki67 in the prostate tissue. The prostates of Pten knockout offspring of HF/HS-fed dams had significantly more pHH3-positive cells than did those from Pten knockout offspring of Chow-fed dams (Fig 4A and 4B). In the Pten WT mice, the HF/HS diet exposed offspring had a significantly higher number of proliferating cells in their prostates than their Chow exposed counterparts (Fig 4B). This data suggests that exposure to maternal HF/HS diet has an additive effect on Pten loss as it increases pHH3 positive cells in both the WT and Pten KO mice. Ki67 positively cells were significantly higher in the Pten KO animals compared to the WT (Fig 4A and 4C). However, we found no significant difference in number of Ki67-positive cells within the two groups in regards to the maternal diet exposure (Fig 4C). These results showed some evidence that proliferation was elevated in mice genetically disposed to prostate cancer and exposed to a maternal diet high in fat and sugar (See S4 and S5 Files).

### HF/HS diet does not have additive effects on pAkt and Akt expression in Pten KO mice

**Fig 5:**
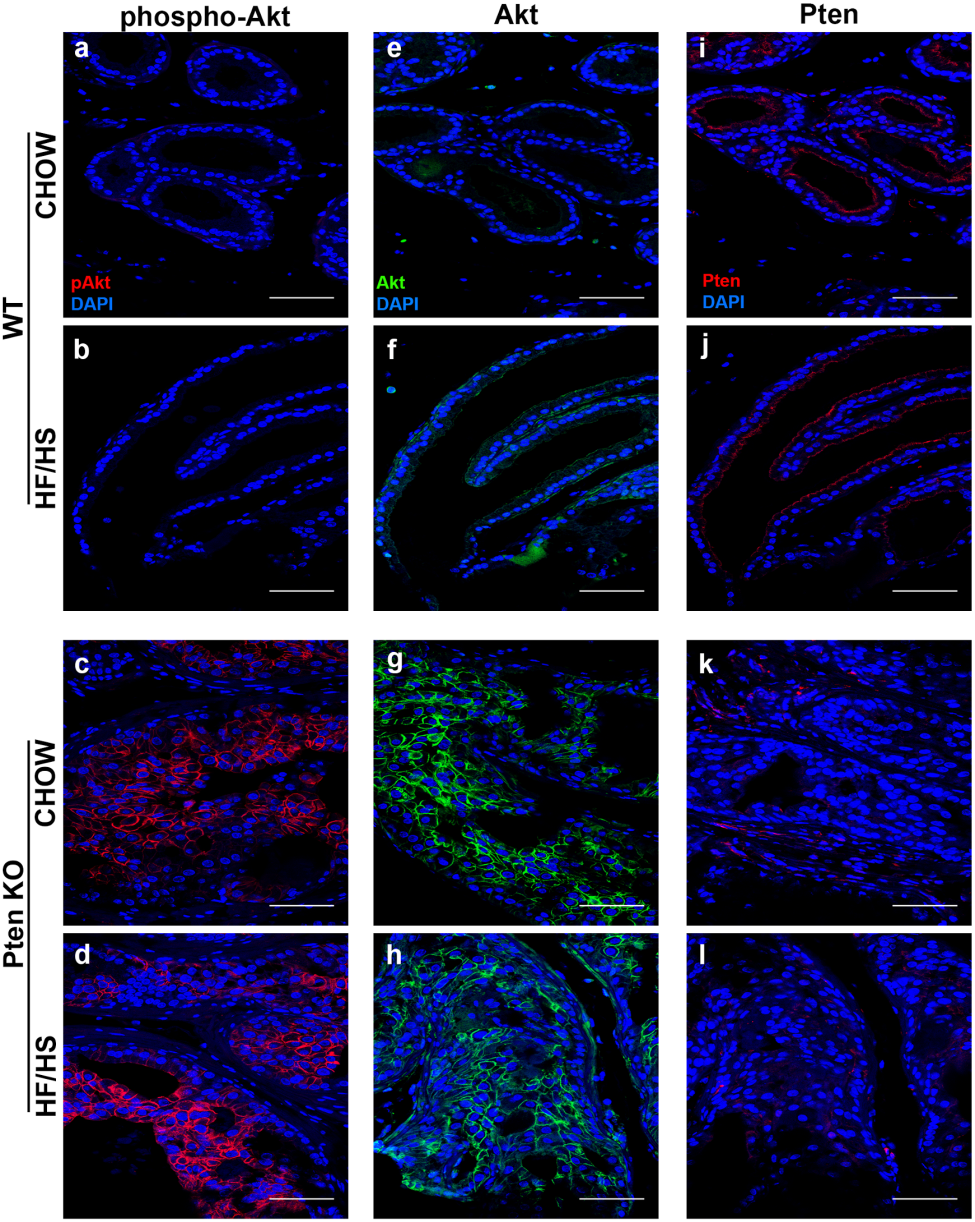
HF/HS diet exposure does not affect phospho-Akt and Akt expression in Pten knockout males. Representative images of phospho-Akt (Ser-473) (a-d), Akt (e-h) and Pten (i-l) staining of prostates from WT and Pten knockout mice exposed to maternal Chow or HF/HS diet. Red—Pten, phospho-Akt, Green—Akt, Blue—DAPI, scale bar = 50 μm.

Activation of Akt occurs through its phosphorylation at serine 473 and results from loss of PTEN. We previously showed that maternal HF/HS diet led to a decrease in Pten expression and a concurrent increase in Akt activation in prostates of 63-week-old male offspring. In this model, we detected an increase in both phospho-Akt (Fig 5C and 5D) and total Akt (Fig 5G and 5H) with the loss of Pten (Fig 5K and 5L) in Pten KO mice but there was no additive effect of the HF/HS diet on phospho-Akt and Akt expression (Fig 5D and 5H versus Fig 5C and 5G).

### Pten knockout males exhibit features of neuroendocrine differentiation

**Fig 6:**
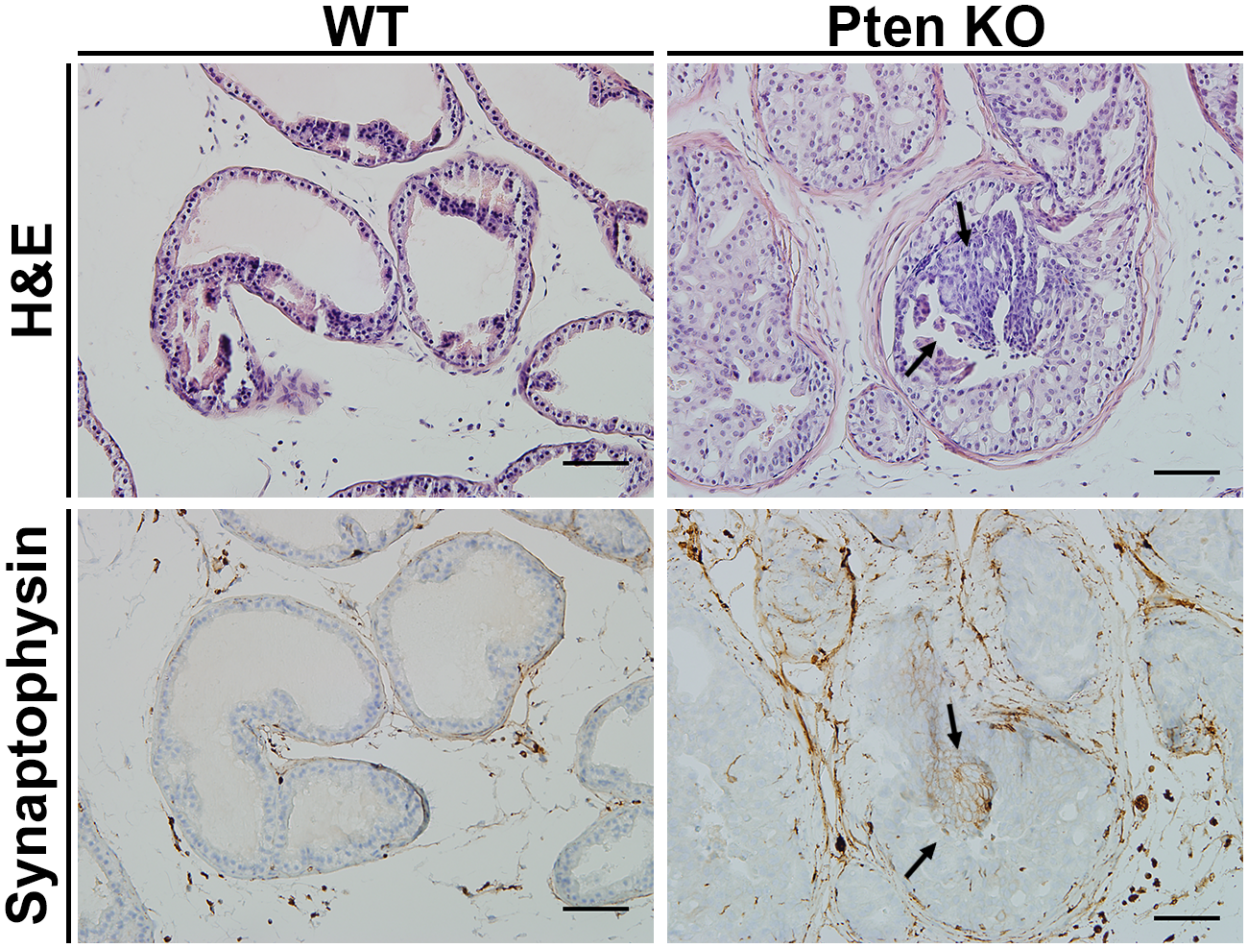
Features of neuroendocrine differentiation observed in Pten kockout males. Representative H&E and synaptophysin staining of prostates from WT and Pten knockout. The WT and Pten knockout mice exposed to maternal HF/HS diet showed similar findings as their Chow counterparts and these findings are summarized in Table 1. Black arrows show areas of NED. Scale bar = 100 μm.

During our histological analysis, we noticed that some of the prostates from Pten knockout mice showed focal areas with subtle features suspicious for neuroendocrine differentiation (NED), such as amphophilic cytoplasm, cell crowding, trabecular growth, and hyperchromatic, speckled chromatin (Fig 6, black arrows). To further assess this effect, we stained the prostate tissue with an antibody specific to the NED marker synaptophysin. The synaptophysin marker showed focal positive immunohistochemical staining in areas correlating with the morphologic impressions of NED. We quantified NED by categorizing synaptophysin-stained sections as negative, equivocal, or positive for NED. We found no difference in the presence of NED between Pten offspring of HF/HS- and Chow-fed dams (Table 2). These data suggest that exposure to maternal HF/HS diet did not enhance the NED phenotype in this mouse model. Nevertheless, NED is a new feature of the Pten knockout prostate cancer mouse model that had not been previously described (See S6 File).

**Table 2:** Summary of mice exhibiting neuroendocrine differentiation as established by positive synaptophysin staining.

		Synaptophysin Staining			
		Negative	Equivocal	Positive	Total
**WT (n, %)**	**Chow and HF/HS**	6, 100%	0	0	6
**Pten KO (n, %)**	**Chow**	2, 28.6%	2, 28.6%	3, 42.8%	7
	**HF/HS**	0	1, 20%	4, 80%	5

## Discussion

Our previous studies demonstrated that exposure to maternal HF/HS induced prostate hyperplasia in wild-type mice[14]. Here, we demonstrated that maternal HF/HS exposure has a synergistic effect on proliferation in Pten kockout male offspring but overall does not influence mPIN progression to a more advanced disease stage. Additionally, we found that Akt activation was not further increased in Pten knockouts born to HF/HS-fed dams. Finally, we identified a new phenotype, neuroendocrine differentiation, in prostates of Pten knockout mice.

In 2016, prostate cancer was the most commonly diagnosed cancer in men in the Western world, but few modifiable risk factors exist for this disease. Many studies have examined the association between obesity and prostate cancer incidence, producing conflicting results. However, maternal energetics during periconception and gestation has barely been considered as a potential risk factor, despite the fact that prostate tissue patterning and early development occur in utero. Maternal gestational obesity influences the risk of several chronic conditions in offspring as they become adults, including metabolic syndrome, cardiometabolic disease, neurodevelopmental disorders[94], impaired circadian rhythms of the hypothalamus and adipose tissues [95, 96], and asthma [97]. Many cancers are initiated by gene expression changes in cells, environmental modifications that provide fitness to cells in particular niches, or both.

Given that the patterning of tissue expression patterns can be negatively influenced by maternal energetics during periconception, maternal energetic imbalance could also lead to changes, such as epigenetic and metabolic defects, that predispose tissues to becoming cancerous.

Few studies have investigated maternal obesity as a risk factor for the development of cancers in offspring despite the fact that patient’s BMI influences the outcomes of many cancers, including advanced prostate adenocarcinoma. Additionally, identification of modifiable risk factors for these malignancies is critical so as to develop preventative interventions. As early development is essential for the patterning of tissue behaviors that will persist throughout the life of the offspring (e.g.,inflammation and metabolism), this time window is a prime candidate. However, prospective studies of early developmental exposures that may increase risk for cancer in adulthood would be extremely costly and challenging in humans. Retrospective studies are feasible, but they suffer from the fact that recall (e.g., asking 60-year-old prostate cancer patients how much their mothers weighed during pregnancy) at the time of diagnosis is unreliable. Nonetheless, some studies have been done in human and animal models to address whether the maternal energetics is a modifiable risk factor for cancer and which time window is the appropriate target for potential interventions.

In the analysis of our model in this work, our histological findings and pHH3 staining were consistent with one another. This is logical since phosphorylation of histone H3 occurs during late G2 and mitosis, and the anti-pHH3 antibody recognizes metaphase chromosomes[24, 25]. However, our quantitation of Ki67 staining did not reveal a significant difference between prostates from HF/HS- and Chow-exposed mice. This may be due to the fact that Ki67 is expressed in all cells during all active phases of the cell cycle and is associated with ribosomal RNA transcription[26]. The cells detected by Ki67 in our images could be proliferative lymphocytes, endothelial cells or stromal cells[27], and thus pHH3 is a more reliable indicator of proliferation in this case. It is also possible that prostate cells in HF/HS-exposed male mice underwent metaphase arrest or stalling, which would explain why we observed an increased number of mitotic figures. These findings suggest that maternal HF/HS diet increases proliferation in the prostate tissue in male offspring.

We previously showed that the PI3K-AKT-mTOR pathway in prostates was affected by exposure to maternal HF/HS diet. Because this pathway is essential for NED, and activation of AKT by factors such as IGF-1 promotes NED[28–30], we assessed whether loss of Pten would synergize with HF/HS diet exposure to increase NED. We were especially interested in NED because it correlates with prostate tumor grade and poor prognosis in humans[17]. Normal prostate consists of three cell types: luminal, basal and neuroendocrine (NE) cells. In human prostate cancer, NE tumor cells are present in about 5–10% of adenocarcinomas, in some PIN cases, and in metastatic disease[31–34]. However, small cell carcinoma of the prostate, which occurs in 0.5 to 2% of prostate cancers, is derived from NE cells[29]. The phenotype that we observed suggests that the developing prostate tumors were not of NE cell origin but had focal NED features[35]. We did not see a significant difference in NED between Chow- and HF/HS-exposed Pten knockouts. However, this is the first description of NED in these Pten knockout mice and could be an essential finding for prostate cancer patients with PTEN mutations.

Work from Kwon et al., showed that diet high in fat promoted the formation of sporadic mPIN lesions in mice[36]. Further, our previous work showed that maternal diet led to prostate hyperplasia at 63 weeks with significant proliferation detected as early as 26 weeks[14]. In our current model we did not observe additive effects of maternal HF/HS diet to Pten loss in the mouse prostate. It is possible that while exposure to maternal HF/HS diet independently affects prostate health in the offspring, in the Pten knockout mouse model, the complete loss Pten dwarfs any additional effects that might be observed due to the diet. An important next question will be to determine how maternal HF/HS exposure can worsen prostate phenotypes in offspring in a less aggressive model of prostate cancer. This could be done in Pten heterozygous mouse model that has a longer latency and slower progression to prostate cancer. This type of model will allow for a comprehensive examination of the effects of maternal HF/HS diet on offspring. We have previously shown that metabolic dysfunction in the mother can be transmitted to the F3 generation and is associated with inheritance of dysfunctional mitochondria[37]. Another possibility is that HF/HS exposure causes epigenetic modifications that result in gene expression changes. Future work should be directed at exploring these possibilities.

## Supporting information

S1 Fig Representative pHH3 and Ki67 staining of prostates from Pten wildtype (WT) mice exposed to maternal Chow or HF/HS diet. Red—pHH3, Green—Ki67, Blue—DAPI (nuclei). Scale bars = 25 μm.
(TIF)

S1 File Data file on body weights, body composition and glucose tolerance in animals from Fig 2. (ZIP)

S2 File Data file on ducts with expansile central necrosis and mitotic figures from Fig 3. (XLSX)

S3 File Additional data file on statistics from Fig 3. (DOCX)

S4 File Data file on Ki67 and positive staining from Fig 4. (PZFX)

S5 File Data file on mitotic figures from Fig 3. (XLSX)

S6 File Data file on PTEN mice from Figs 3 and 6. (XLSX)
