# Supplementary figures and images for: Exposure to maternal obesogenic diet worsens some but not all pre-cancer phenotypes in a murine genetic model of prostate cancer
Source: PLoS One. 2017 May 10;12(5):e0175764. doi: 10.1371/journal.pone.0175764 (PMC5425180; doi:10.1371/journal.pone.0175764)

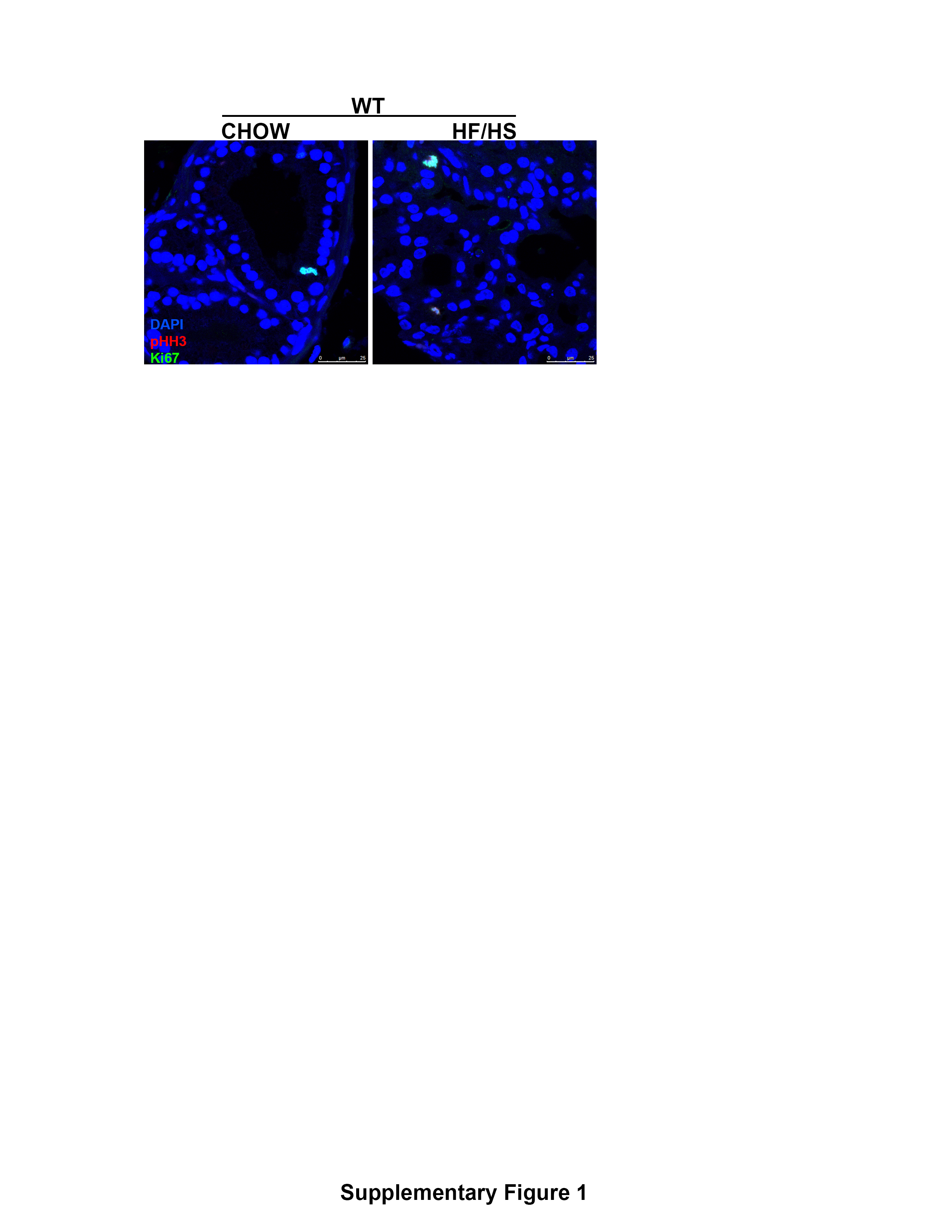

Supplement: S1 Fig — Red—pHH3, Green—Ki67, Blue—DAPI (nuclei). Scale bars = 25 μm. (TIF) [file pone.0175764.s001.tif]
