# Supplementary material for: Exposure to maternal obesogenic diet worsens some but not all pre-cancer phenotypes in a murine genetic model of prostate cancer
Source: PLoS One. 2017 May 10;12(5):e0175764. doi: 10.1371/journal.pone.0175764 (PMC5425180; doi:10.1371/journal.pone.0175764)

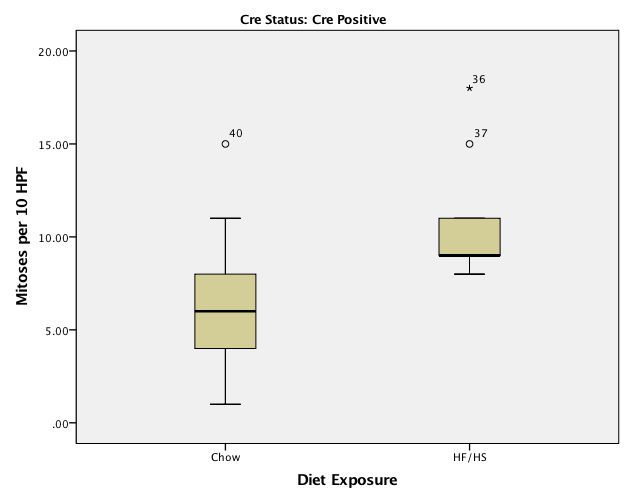


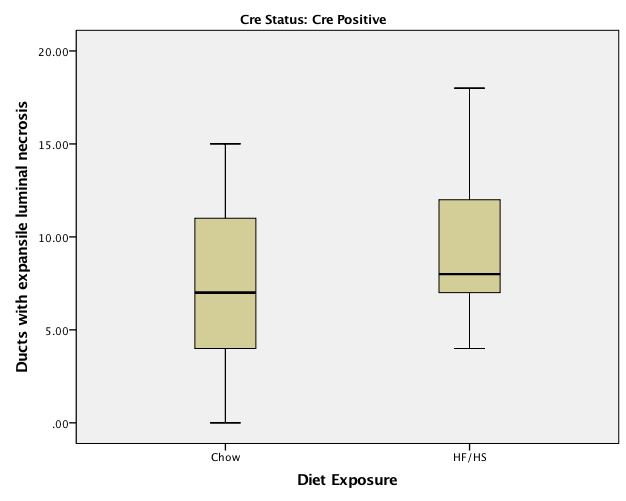


| **Group Statistics** | | | | | | |
| --- | --- | --- | --- | --- | --- | --- |
| Cre Status | | Diet Exposure | N | Mean | Std. Deviation | Std. Error Mean |
| Cre Negative | Mitoses per 10 HPF | Chow | 10 | 1.1000 | .99443 | .31447 |
|  |  | HF‎/HS | 12 | .5833 | .79296 | .22891 |
|  | Ducts with expansile luminal necrosis | Chow | 10 | .0000 | .00000^a^ | .00000 |
|  |  | HF‎/HS | 12 | .0000 | .00000^a^ | .00000 |
| Cre Positive | Mitoses per 10 HPF | Chow | 13 | 6.5385 | 3.75534 | 1.04154 |
|  |  | HF‎/HS | 9 | 10.7778 | 3.45607 | 1.15202 |
|  | Ducts with expansile luminal necrosis | Chow | 13 | 7.1538 | 4.59794 | 1.27524 |
|  |  | HF‎/HS | 9 | 9.8444 | 5.06140 | 1.68713 |
| a. t cannot be computed because the standard deviations of both groups are 0. | | | | | | |


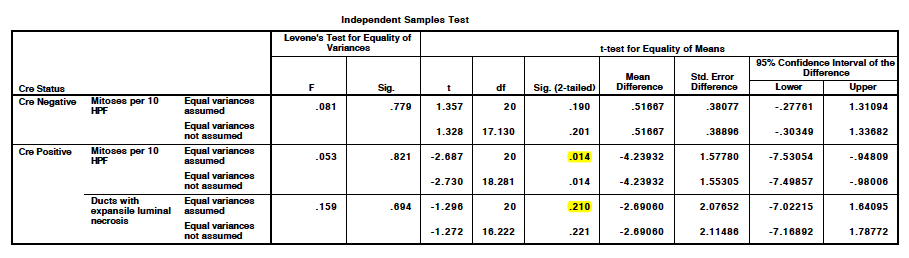

Supplement: S3 File — (DOCX) [file pone.0175764.s004.docx]
